# Single nucleotide polymorphisms as prognostic and predictive biomarkers in renal cell carcinoma

**DOI:** 10.18632/oncotarget.22533

**Published:** 2017-11-20

**Authors:** Carmen Garrigós, Marta Espinosa, Ana Salinas, Ignacio Osman, Rafael Medina, Miguel Taron, Sonia Molina-Pinelo, Ignacio Duran

**Affiliations:** ^1^ Instituto de Biomedicina de Sevilla, IBiS, Hospital Universitario Virgen del Rocío, CSIC, Universidad de Sevilla, Sevilla, Spain; ^2^ Unidad de Urología Oncológica, UGC Urología-Nefrología H.U.Virgen del Rocío, Instituto de Biomedicina de Sevilla, IBiS, Hospital Universitario Virgen del Rocío, CSIC, Universidad de Sevilla, Sevilla, Spain; ^3^ Centro de Investigación Biomédica en Red Cáncer, CIBERONC, Madrid, Spain

**Keywords:** single nucleotide polymorphisms, angiogenesis genes, biomarkers, localized renal cell carcinoma, advanced renal cell carcinoma

## Abstract

Despite major advances in the knowledge of the molecular basis of renal cell carcinoma, prognosis is still defined using clinical and pathological parameters. Moreover, no valid predictive biomarkers exist to help us selecting the best treatment for each patient. With these premises, we aimed to analyse the expression and to determine the prognostic and predictive value of 64 key single nucleotide polymorphisms in 18 genes related with angiogenesis or metabolism of antiangiogenics in two cohorts of patients with localized and advanced renal cell cancer treated at our institution. The presence of the selected single nucleotide polymorphisms was correlated with clinical features, disease free survival, overall survival and response rate. In patients with localized renal cell cancer, 5 of these polymorphisms in 3 genes involved in angiogenesis predicted for worse disease free survival (VEGFR2: rs10013228; PDGFRA: rs2228230) or shorter overall survival (VEGFR2: rs10013228; VEGFR3: rs6877011, rs307826) (*p* < 0.05). Rs2071559 in VEGFR2 showed a protective effect (*p* = 0.01). In the advanced setting, 5 SNPs determined inferior overall survival (IL8: rs2227543, PRKAR1B: rs9800958, PDGFRB: rs2302273; *p* = 0.05) or worse response rate (VEGFA: rs699947, rs3025010 *p* ≤ 0.01)). Additionally 1 single nucleotide polymorphism in VEGFB predicted for better response rate rs594942 (*p* = 0.03). Genetic analysis of renal cell carcinoma patients might provide valuable prognostic/predictive information. A set of SNPs in genes critical to angiogenesis and metabolism of antiangiogenics drugs seem to determine post-surgical outcomes and treatment response in our series.

## INTRODUCTION

Renal cell carcinoma (RCC) is the most common malignancy of the kidney with near 338.000 new diagnoses per year worldwide [1]. It is more frequent in men and 75% of the patients are diagnosed over 60 years of age. Incidence of RCC has increased steadily at 2% per year contributing to about 144.000 deaths in 2012 [2, 3]. Diverse histological variants have been described including clear cell (75%), papillary (10%), chromophobe (5%) and others [4]. Approximately 25% of the patients present with advanced disease at diagnosis, and up to one third of those with localized disease that undergo surgery with a curative intention will recur requiring systemic treatment [5].

A better understanding of the molecular biology of RCC has allowed remarkable progress in therapeutics in the last decade. This advance comes primarily from the description of the Von Hippel -Lindau (VHL) syndrome; a hereditary condition associated with a mutation in the homonymous tumor suppressor gene, in which around 60% of the patients develop clear cell RCC (ccRCC). In normal conditions the VHL product (VHLp) creates a complex that targets hypoxia inducible factors 1 and 2 (HIF 1-2) for ubiquitin-mediated degradation. In the absence of VHLp by either mutation or methylation of VHL gene, HIF accumulates leading to exaggerated transcription of multiple genes involved in cell proliferation and angiogenesis such as the platelet-derived growth factor (PDGF), vascular endothelial growth factor (VEGF) and transforming growth factor [6–9]. The VEGF binds its receptor (VEGFR) and promotes proliferation and migration of endothelial cells, increased vascular permeability and revascularization during tumor development [10–12]. Similarly, PDGF and its receptors (PDGFRA, PDGFRB) play a critical role in regulating angiogenesis through controlling functions during the mesenchymal cell development. Signalling through PDGF also promotes cell migration, survival and proliferation and indirectly regulates angiogenesis by inducing transcription and secretion of VEGF [13]. These knowledge and the observation that around 90% of sporadic ccRCC have abnormal function of VHL has led to an intense drug development in RCC targeting VEGF, PDGF or their cognate receptors. Bevacizumab, a humanized monoclonal antibody against VEGF, was the first agent in this class to demonstrate activity in advanced RCC [14]. Thereafter multiple antiangiogenics such as the tyrosine kinase inhibitors sunitinib, sorafenib, pazopanib or axitinib and mTOR inhibitors such as temsirolimus or everolimus, have shown remarkable activity in advanced RCC becoming standard of treatment in different settings [15]. More recently other therapeutic strategies such as targeting the program-death 1 (PD-1) receptor or the hepatocyte growth factor receptor (MET) have also succeeded [16, 17].

Although the availability of all these drugs has improved substantially the therapeutic results in RCC, approximately 40% of patients treated in first-line will not achieve an objective response and about 20–25% will present an early progression. Currently available prognostic systems fail to identify these patients and no adequate predictive factors of response have been validated in advanced RCC yet.

The variability in the genetic constitution of the individual in critical genes related to disease mechanisms or anti-cancer drug metabolism could explain this variable clinical course. Single nucleotides polymorphisms (SNPs) are the most common genetic variations in the DNA sequence, involve a single base and have a frequency of greater than 1% in at least one minor allele population [18]. Certain SNPs have already been identified as potential predictors of efficacy and/or toxicity in advanced RCC patients treated with tyrosine kinase inhibitors [19–26].

The present study aims to analyse the incidence of SNPs in genes related with angiogenesis or metabolism of antiangiogenics in patients with localized and advanced RCC and to test their potential as prognostic and/or predictive factors.

## RESULTS

One hundred and two patients were initially included in the study, 65% were male and the median age was 62 years (range 29–83 years). Three patients were excluded from the final analysis due to incomplete clinical information available. The median of follow-up was 62 months. Table 1A shows clinical characteristics for localized (a) and metastatic (b) patients and the association of these characteristics with disease/progression free survival (DFS and PFS) and overall survival (OS) (c).

**Table 1A:** Clinical features of patients with localized disease. *N* = number of patients, % = percentage of patients, Total = total of patients

Clinical feature	*N*	%	Total
**Gender**			
Male	45	62%	73
Female	28	38%	
**Age**			
≥60	45	62%	73
<60	28	38%	
**Diagnosis**			
Incidental	36	49%	73
Back Pain	1	1%	
Hematuria	8	11%	
Constitutional symptoms	4	6%	
Others	24	33%	
**Nephrectomy**			
Yes	73	100%	73
No	**–**	**–**	
Partial	3	4%	73
Complete	70	96%	
Open surgery	64	88%	73
Laparoscopy	9	12%	
**Histology**			
Clear cell	54	74%	73
Papillary	16	22%	
Other	3	4%	
**Furhman Grade**			
1	24	36%	66^*^
2	21	32%	
3	18	27%	
4	3	5%	
**Furhman Grade Groups**			
1–2	46	70%	66^*^
3–4	20	30%	
**Diagnosis TNM**			
T1–T2	54	74%	73
T3–T4	19	26%	
**ECOG**			
0	20	83%	24^*^
>0	4	17%	

^*^Only available data is presented.

**Table 1B:** Clinical features of patients with metastatic disease. *N* = number of patients, % = percentage of patients, Total = total of patients

Clinical feature	*N*	%		Total
**Gender**				
Male	35	70%		50
Female	15	30%		
**Age**				
≥60	22	44%		50
<60	28	66%		
**Diagnosis**				
Incidental	15	30%		50
Back Pain	2	4%		
Hematuria	8	16%		
Constitutional symptoms	6	12%		
Others	19	38%		
**Nephrectomy**				
Yes	43		86%	50
No	7		14%	
Partial	1		2%	43
Complete	42		98%	
Open surgery	39		91%	43
Laparoscopy	4		9%	
**Histology**				
Clear cell	41		82%	50
Papillary	5		10%	
Other	4		8%	
**Furhman Grade**				
1	7		18%	38^*^
2	12		32%	
3	13		34%	
4	6		16%	
**Furhman Grade Groups**				
1–2	19		50%	38^*^
3–4	19		50%	
**Diagnosis TNM**				
T1–T2	15		30%	50
T3–T4	35		70%	
**ECOG**				
0	33		66%	50
>0	17		34%	
**Metastasis**				
Lung	34		68%	50
Liver	7		14%	
Nodes	16		32%	
Bones	6		12%	
Brain	1		2%	
**MSKCC prognosis (Karnofsky Hemoglobin, LDH, calcium)**				
**Karnofsky**				
≥80%	45		90%	50
<80%	5		10%	
**Hemoglobin**				
<LLN	22		44%	50
Normal	28		56%	
**LDH**				
≥1.5 ULN	2		4%	50
<1.5 ULN	48		96%	
**Corrected calcium**				
≥10 mg/dl	1		2%	50
<10 mg/dl	49		98%	
**Time nephrectomy-systemic treatment**				
≥1 year	21		42%	50
<1 year	29		58%	
**Prognosis Group**				
Favorable	12		24%	50
Intermediate	30		60%	
Poor	8		16%	
**Systemic treatment**				
TKI	43	86%		50
Sunitinib	33			
Pazopanib	10			
mTOR	7	14%		
Temsirolimus	5			
Everolimus	2			

^*^Only available data is presented.

**Table 1C:** Clinical features of patients associated with Disease Free Survival (DFS) or Progression Free Survival (PFS) and Overall Survival (OS) ( *p* values > 0.999 not shown)

Patients	Localized disease ( *p*)		Metastatic disease ( *p*)	
Clinical features	DFS	OS	PFS	OS
Gender	0.345	0.767	0.493	0.470
Age (≥60 vs <60)	0.440	0.570	0.108	0.773
Diagnosis (incidental vs others)	0.499	0.832	–	–
Nephrectomy (yes/no)	NA		0.660	0.080
Partial/Complete	0.230	–	–	0.395
Open surgery/ Laparoscopy	0.900	0.426	0.563	0.140
Histology (clear cell vs papillary vs others)	0.997	0.491	0.381	0.168
Furhman Grade (1–2 vs 3–4)	0.185	0.328	0.027	–
TNM (T1–T2 vs T3–T4)	0.001	0.055	0.170	0.474
ECOG (0 vs >0)	–	0.064	0.173	0.017
Metastasis				
Lung			0.179	0.118
Liver			–	0.210
Nodes			0.731	0.357
Bones			–	0.083
Brain			–	–
Karnofsky (≥80% vs <80%)	NA		0.309	0.152
Hemoglobin (<LLN vs normal)			0.339	0.036
LDH (≥1.5 ULN vs <1.5 ULN)			–	0.542
Corrected calcium (≥10 vs <10 mg/dl)			–	–
Time nephrectomy-systemic treatment (≥1 vs <1 year)			0.027	0.020
Prognosis Group (Favorable vs Intermediate/Poor)			0.01	0.001
Systemic treatment (TKI vs mTor)			–	0.235

NA: Not apply for patients with localized disease.

One triallelic SNP (rs2032582) was excluded from the analysis due to inconsistent results with the array utilized. The minor allele frequencies (MAF) of the others 62 polymorphisms genotyped (Table 2) were consistent with the data described elsewhere for European and Iberian population (1000 genomes, dbSNP database) and all SNPs were in Hardy-Weinberg equilibrium ( *p >* 0.05). Table 2 shows the characteristics for the 62 polymorphisms genotyped and frequency in our tumor samples in localized and metastatic patients.

**Table 2:** Characteristics for the 62 polymorphisms genotyped and frequency in our tumor samples in localized and metastatic patients

	dbSNP	Gene	Chrom.	HGVS name	Location	Type of SNP variant	Minor allele	*N* Localized	*N* Metastatic
1	rs699947	VEGFA	6p21.1	6:g.43736389A>C	6:43736389	Upstream gene	A (46.7)	43 (58.9)	16 (61.5)
2	rs833061	VEGFA	6p21.1	6:g.43737486C>T	6:43737486	Upstream gene	C (47.2)	41 (56.2)	16 (61.5)
3	rs3025010	VEGFA	6p21.1	6:g.43747577T>C	6:43747577	Non coding transcript exon	C (36)	36 (49.3)	17 (65.4)
4	rs3025033	VEGFA	6p21.1	6:g.43751075A>G	6:43751075	Non coding transcript exon	G (15)	22 (30.1)	8 (30.8)
5	rs2010963	VEGFA	6p21.1	6:g.43738350C>G	6:43738350	5 prime UTR	C (33.6)	37 (50.7)	11 (42.3)
6	rs1570360	VEGFA	6p21.1	6:g.43737830A>G	6:43737830	Upstream gene	A (30)	15 (20.5)	1 (3.8)
7	rs3025039	VEGFA	6p21.1	6:g.43752536C>T	6:43752536	3 prime UTR	T (13)	15 (20.5)	6 (23.1)
8	rs4930152	VEGFB	11q13.1	11:g.64005412G>A	11:64005412	Intron variant	A (31)	37 (50.7)	69.2 (18)
9	rs594942	VEGFB	11q13.1	11:g.64006292T>C	11:64006292	Upstream gene	T (30)	45 (61.6)	14 (53.8)
10	rs2016110	VEGFC	4q34.3	4:g.177604081A>G	4:177604081	Intron	A (13)	25 (34.2)	8 (30.8)
11	rs1485766	VEGFC	4q34.3	4:g.177610884T>G	4:177610884	Intron	G (49)	42 (57.5)	12 (46.2)
12	rs11947611	VEGFC	4q34.3	4:g.177611397G>A	4:177611397	Intron	A (46)	32 (43.8)	10 (38.5)
13	rs2877967	VEGFC	4q34.3	4:g.177707602C>T	4:177707602	Intron	C (14)	17 (23.3)	4 (15.4)
14	rs4604006	VEGFC	4q34.3	4:g.177608775T>C	4:177608775	Intron	T (27)	24 (32.9)	12 (46.2)
15	rs2305948	VEGFR2	4q12	4:g.55979558C>T	4:55979558	Missense	T (14)	21 (28.8)	4 (15.4)
16	rs1870377	VEGFR2	4q12	4:g.55972974T>A	4:55972974	Missense	A (25)	21 (28.8)	6 (23.1)
17	rs12505758	VEGFR2	4q12	4:g.55966898T>C	4:55966898	Intron	C (12)	26 (19)	19.2 (5)
18	rs10013228	VEGFR2	4q12	4:g.55997340A>G	4:55997340	Intergenic	G (30)	32 (43.8)	11 (42.3)
19	rs11941492	VEGFR2	4q12	4:g.55978210C>T	4:55978210	Intron	T (22)	25 (34.2)	7 (26.9)
20	rs2071559	VEGFR2	4q12	4:g.55992366A>G	4:55992366	Upstream gene	A (47)	50 (68.5)	16 (61.5)
21	rs1531290	VEGFR2	4q12	4:g.55986562G>A	4:55986562	Intron	A (47)	41 (56.2)	15 (57.7)
22	rs6828477	VEGFR2	4q12	4:g.55966801C>T	4:55966801	Intron	C (43)	18 (24.7)	10 (38.5)
23	rs307826	VEGFR3	5q35.3	5:g.180051003T>C	5:180051003	Missense	C (12)	9 (12.3)	5 (19.2)
24	rs307821	VEGFR3	5q35.3	5:g.180030313C>A	5:180030313	Missense	A (11)	8 (11)	1 (3.8)
25	rs6877011	VEGFR3	5q35.3	5:g.180029471C>G	5:180029471	3 prime UTR	G (6)	6 (8.2)	5 (19.2)
26	rs779805	VHL	3p25.3	3:g.10183337G>A	3:10183337	5 prime UTR	G (28)	25 (34.2)	9 (34.6)
27	rs1642742	VHL	3p25.3	3:g.10191943G>A	3:10191943	3 prime UTR	G (29)	28 (38.4)	15 (57.7)
28	rs2227543	IL8	4q13.3	4:g.74607910C>T	4:74607910	3 prime UTR	T (42)	37 (50.7)	14 (53.8)
29	rs4073	IL8	4q13.3	4:g.74606024A>T	4:74606024	Upstream gene	A (47)	30 (41.1)	11 (42.3)
30	rs1800795	IL6	7p15.3	7:g.22766645C>G	7:22766645	Intron	C (35)	36 (49.3)	17 (65.4)
31	rs1045642	ABCB1	7q21.12	7:g.87138645A>T	7:87138645	Synonymous	A (46)	46 (63)	20 (76.9)
32	rs1128503	ABCB1	7q21.12	7:g.87179601A>G	7:87179601	Synonymous	A (38)	51 (69.9)	18 (69.2)
33	rs2231142	ABCG2	4q22.1	4:g.89052323G>T	4:89052323	Missense	T (7)	5 (6.8)	3 (11.5)
34	rs3814055	NR1I2	3q13.33	3:g.119500035C>T	3:119500035	5 prime UTR	T (40)	47 (64.4)	15 (57.7)
35	rs2276707	NR1I2	3q13.33	3:g.119534153C>T	3:119534153	Intron	T (18)	21 (28.8)	13 (50)
36	rs2307424	NR1I3	1q23.3	1:g.161202605G>A	1:161202605	Synonymous	A (35)	32 (43.8)	16 (61.5)
37	rs4073054	NR1I3	1q23.3	1:g.161200487C>A	1:161200487	3 prime UTR	C (34)	34 (46.6)	11 (42.3)
38	rs2740574	CYP3A4	7q22.1	7:g.99382096C>T	7:99382096	Upstream gene	C (3)	3 (4.1)	1 (3.8)
39	rs776746	CYP3A5	7q22.1	7:g.99270539C>T	7:99270539	Splice acceptor	T (7)	9 (12.3)	4 (15.4)
40	rs9800958	PRKAR1B	7p22.3	7:g.668723A>G	7:668723	Intron	A (27)	59 (80.8)	20 (76.9)
41	rs9768991	PRKAR1B	7p22.3	7:g.671687T>C	7:671687	Intron	T (27)	25 (34.2)	9 (34.6)
42	rs9611117	PDGFB	22q13.1	22:g.39624105T>G	22:39624105	Intron	G (45)	51 (69.9)	14 (53.8)
43	rs879180	PDGFB	22q13.1	22:g.39631547T>C	22:39631547	Intron	T (26)	26 (35.6)	11 (42.3)
44	rs35597368	PDGFRA	4q12	4:g.55139771T>C	4:55139771	Missense	C (8)	8 (11)	7 (26.9)
45	rs2114039	PDGFRA	4q12	4:g.55092626T>C	4:55092626	Intron	C (30)	28 (38.4)	9 (34.6)
46	rs6554162	PDGFRA	4q12	4:g.55093955G>A	4:55093955	Intron	A (30)	31 (42.5)	11 (42.3)
47	rs1800812	PDGFRA	4q12	4:g.55094629G>T	4:55094629	Intron	T (20)	20 (27.4)	7 (26.9)
48	rs4358459	PDGFRA	4q12	4:g.55133726T>G	4:55133726	Synonymous	G (10)	9 (12.3)	6 (23.1)
49	rs2228230	PDGFRA	4q12	4:g.55152040C>T	4:55152040	Synonymous	T (13)	12 (16.4)	4 (15.4)
50	rs17739921	PDGFRA	4q12	4:g.55164866A>C	4:55164866	Downstream gene	C (47)	51 (69.9)	15 (57.7)
51	rs246395	PDGFRB	5q32	5:g.149499672T>C	5:149499672	Synonymous	C (27)	44 (60.3)	22 (84.6)
52	rs246394	PDGFRB	5q32	5:g.149498151G>A	5:149498151	Intron	A (25)	35 (47.9)	10 (38.5)
53	rs3816018	PDGFRB	5q32	5:g.149508475C>T	5:149508475	Intron	C (44)	39 (53.4)	15 (57.7)
54	rs17708574	PDGFRB	5q32	5:g.149521238G>A	5:149521238	Intron	A (16)	13 (17.8)	9 (34.6)
55	rs2302273	PDGFRB	5q32	5:g.149535255G>A	5:149535255	5 prime UTR	A (24)	33 (45.2)	6 (23.1)
56	rs3828610	PDGFRB	5q32	5:g.149535625A>C	5:149535625	Upstream gene	C (41)	39 (53.4)	10 (38.5)
57	rs2304060	PDGFRB	5q32	5:g.149501751A>C	5:149501751	Non coding transcript exon	C (43)	47 (64.4)	17 (65.4)
58	rs17656204	PDGFRB	5q32	5:g.149501803C>T	5:149501803	Intron	T (26)	38 (52.1)	13 (50)
59	rs11748255	PDGFRB	5q32	5:g.149512042G>A	5:149512042	Intron	A (48)	43 (58.9)	18 (69.2)
60	rs11740355	PDGFRB	5q32	5:g.149513626T>G	5:149513626	Non coding transcript exon	G (8)	6 (8.2)	2 (7.7)
61	rs3776081	PDGFRB	5q32	5:g.149532107T>C	5:149532107	Intron	C (37)	40 (54.8)	14 (53.8)
62	rs4324662	PDGFRB	5q32	5:g.149531111C>T	5:149531111	Intron	T (24)	31 (42.5)	8 (30.8)

Chrom: Chromosome, Minor allele frequency for European or Iberian population (%). *N*: number of patients with the minor allele frequency (%).

Upstream gene: the sequence variant is located in the 5′ position of the gene.

Downstream gene: the sequence variant is located in the 3′ position of the gene.

Patients were classified in two cohorts for analysis purposes: localized and metastatic. A number of SNPs showed either a protective or adverse effect (Table 3A). Thus, in patients with localized tumors, one polymorphism, rs2071559 in VEGFR2 gene was associated with a protective effect: the mean of patients with this SNP presented a DFS of 49 month vs. 19 months when the SNP was absent. Another two, rs2228230 and rs10013228 in two genes (PDGFRA and VEGFR2) were significantly associated with worse DFS in the multivariate analysis. Accordingly, the absence of rs2228230 associated with an increased DFS (43 months) compared with 25 months in those patients harbouring the SNP. For rs10013228 the deleterious effect in DFS was even of a larger magnitude (62 months vs. 31 months). Additionally, rs10013228 was also significantly associated with a shorter OS (136 vs. 120 months). Other two SNPs (rs307826 and rs6877011) in VEGFR3 were also confirmed as predictors of shorter OS (127 vs. 96 months and 139 vs. 30 months, respectively).

**Table 3A:** Most representative SNPs in patients with localized disease

dbSNP	Gene	DFS [Months (m)]			
		SNP present	UV ( *p*)	MV ( *p*)	HR
rs2071559	VEGFR2	49 vs 19 m	**0.003**	**0.01**	0.2
rs10013228	VEGFR2	31 vs 62 m	0.07	**0.03**	4.6
rs1870377	VEGFR2	23 vs 51 m	**0.03**	0.08	3.5
rs2228230	PDGFRA	25 vs 43 m	0.21	**0.01**	21

dbSNP	Gene	OS [Months (m)]			
		SNP present	UV ( *p*)	MV ( *p*)	HR
rs10013228	VEGFR2	120 vs 136 m	**0.04**	**0.01**	5.5
rs2305948	VEGFR2	103 vs 138 m	**0.04**	0.06	2.9
rs307826	VEGFR3	96 vs 127 m	0.10	**0.03**	3.6
rs6877011	VEGFR3	30 vs 139 m	**0.001**	**0.003**	5.5

DFS: disease free survival, OS: overall survival, UV: univariant analysis, MV: multivariant analysis, HR: hazard ratio.

**Figure 1:**
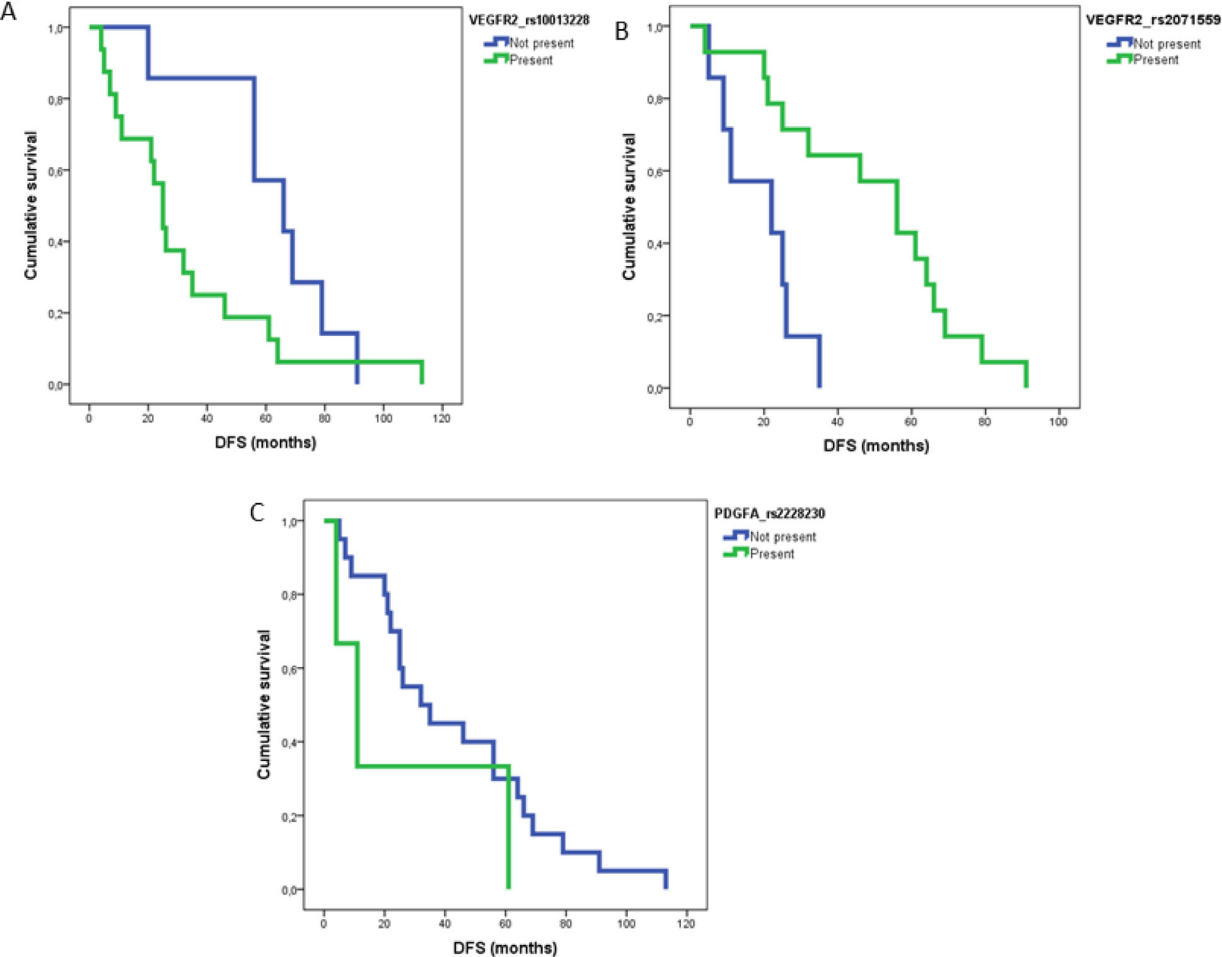
DFS curves for statistically significant SNPs in MV analysis for patients with localized disease (**A**) rs10013228 ( *p* = 0.03); (**B**) rs2071559 ( *p* = 0.01) and (**C**) rs2228230 ( *p* = 0.01).

**Figure 2:**
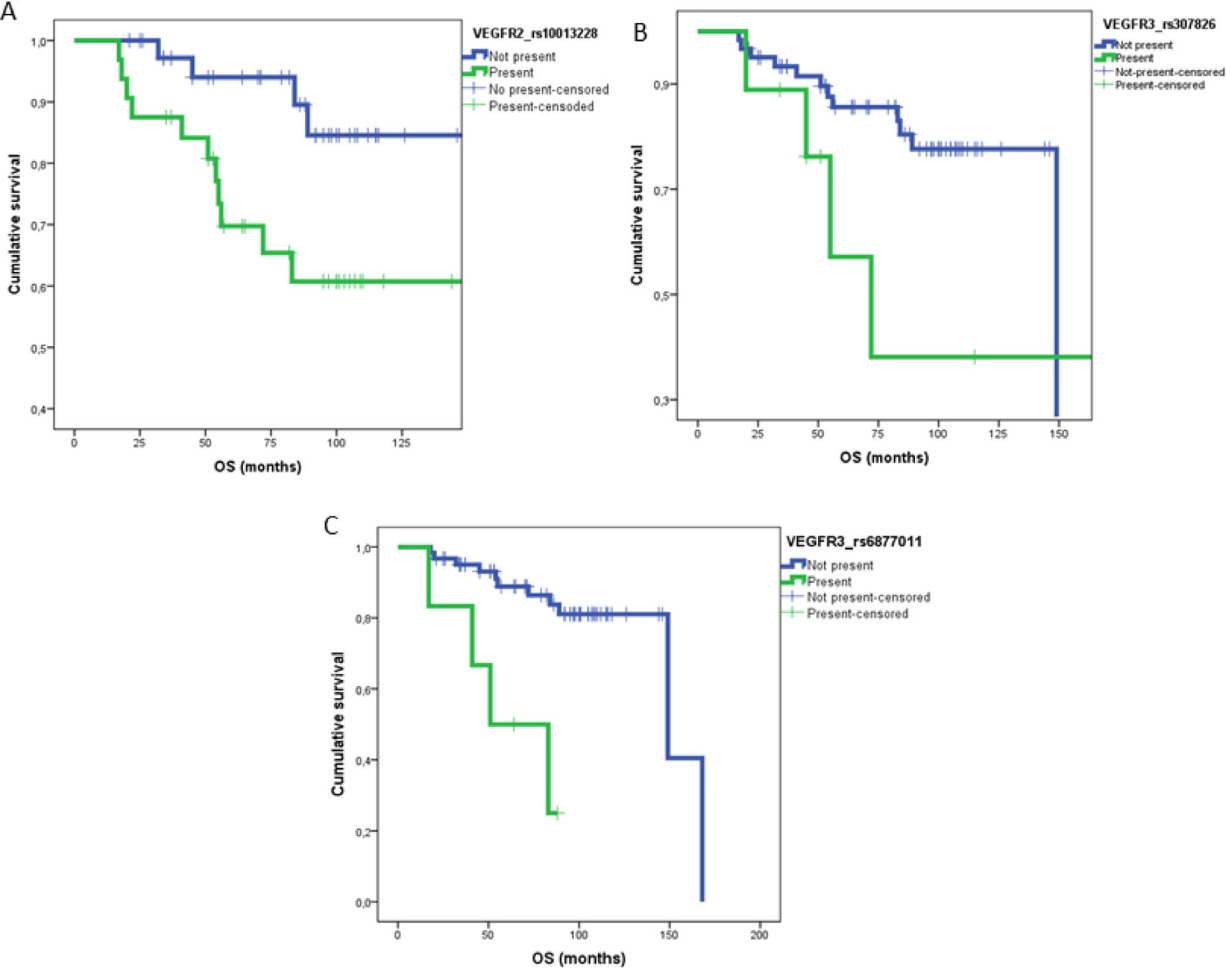
OS curves for statistically significant SNPs in MV analysis for patients with localized disease (**A**) rs10013228 ( *p* = 0.01); (**B**) rs307826 ( *p* = 0.03) and (**C**) rs6877011 ( *p* = 0.003).

In metastatic patients, two SNPs: rs9800958 (PRKAR1B) ( *p =* 0.05) and rs2302273 (PDGFRB) ( *p =* 0.05), showed a tendency towards a better OS in the multivariant analysis (Table 3B). In terms of activity, two SNPs correlated with better response rate (RR): rs2016110 (VEGFC) ( *p =* 0.07) and rs594942 (VEGFB) ( *p =* 0.03) and another two: rs699947and rs3025010 (VEGFA) ( *p <* 0.005) associated with a worse RR (Table 3C). DFS and OS curves for statistically significant SNPs are illustrated in Figures 1 and 2. When analyzing the predictive role of clinical variables, in the localized disease cohort, TNM stage T1 or T2 associated with a better DFS ( *p =* 0.001) and OS ( *p =* 0.055). In metastatic patients, Fuhrman grade (1–2) ( *p =* 0.027) correlated with better progression free survival (PFS) and normal hemoglobin ( *p =* 0.036) and ECOG 0 were significant for a better OS. Intermediate or poor prognosis ( *p* ≤ 0.01) and time between nephrectomy and systemic treatment (˃1 year) ( *p =* 0.020) were associated with both, shorter PFS and OS.

**Table 3B:** Most representative SNPs in patients with metastatic disease

dbSNP	Gene	OS [Months (m)]			
		SNP present	UV ( *p*)	MV ( *p*)	HR
rs9800958	PRKAR1B	32 vs 14 m	**0.03**	0.05	0.3
rs2302273	PDGFRB	42 vs 19 m	**0.014**	0.05	0.1

OS: overall survival, UV: univariant analysis, MV: multivariant analysis, HR: hazard ratio.

**Table 3C:** Most representative SNPs in patients with metastatic disease

dbSNP	Gene	Result SNP present	RR		Total (*n*)	( *p*)	
			Responder (*n*)	No-responder (*n*)		UV	MV
rs2016110	VEGFC	Better prognosis	19	4	23	**0.009**	0.07
rs594942	VEGFB	Better prognosis	16	4	20	**0.025**	**0.03**
rs699947	VEGFA	Worse prognosis	8	23	31	**0.01**	**0.004**
rs3025010	VEGFA	Worse prognosis	7	23	30	**0.009**	**0.002**

RR: response rate, UV: univariant analysis, MV: multivariant analysis. (n): number of patients.

## DISCUSSION

Despite major advances in the knowledge of the molecular basis and therapeutics of RCC, prognostic and predictive estimation remains largely based on clinical and blood test parameters. This is an exploratory pharmacogenetic study designed to identify SNPs that could contribute to select patients with better prognosis and /or higher chances of benefiting from systemic treatment. We studied 62 polymorphisms from 18 genes in 99 patients on the basis of allele frequency and functionality evidence. Our study showed that the presence of certain SPNs was statistically associated with the progression of the disease, the response to treatment and the overall survival in this RCC patient population.

In patients with localized disease, the SNPs that had clinical significance were those positioned in receptors of VEGF and PDGF such as VEGFR2, VEGFR3 or PDGFR. SNPs located in these genes could potentially influence the activation of their cognate signaling pathways, which is a well-established mechanism of RCC tumorigenesis. We found that patients wild type for rs10013228 have a better DFS and OS. No studies in European populations or in RCC patients have been found in this regard. To our knowledge, the only reference in the literature of this SNP comes from a Chinese cohort of localized colorectal cancer patients where it had shown a protective effect [27]. Rs2071559 is a promoter SNP associated with VEGFR2 transcription activity [28]. In our study the AA genotype was associated with a protective effect increasing the DFS. These results are in concordance with data from other reported studies. In a recent metastatic RCC analysis [28], this polymorphism was shown to predict for sorafenib (an anti-VEGFR) efficacy. Promising results have been also described in metastatic colorectal cancer where this VEGFR2 polymorphism was significantly associated with increased PFS and OS in multivariate analysis in metastatic patients treated with first-line oxaliplatin-based chemotherapy regardless the KRAS mutational status [29]. Likewise a study in patients with localized colorectal cancer suggested a protective role for rs2071559, especially in patients that had received chemotherapy [27]. Data from other tumor types also pointed in a similar direction. An analysis in hepatocellular carcinoma patients treated with sorafenib showed that the presence of rs2071559 was a predictor of better outcomes [30]. SNPs in VEGFR3 were also associated with treatment outcomes. Thus, the absence of the SNPs rs307826 and rs6877011 were predictors of better outcome. This is consistent with other reports in RCC patients treated with the anti-VEGFR sunitinib where the presence of the genetic variant rs307826 or rs6877011 was associated with a shorter DFS [19] and OS [31].

Our study also found that SNPs in the PDGFRA gene such as rs2228230 significantly associated with worse prognosis. No previous reports exist about this SNP in RCC. Its presence has been reported in rare cancers such as extra-intestinal stromal tumors and cervical adeno-squamous carcinoma, nevertheless its prognostic or predictive role remains largely unexplored [32, 33]. In our series we could not confirm a variation in response to different PDGFR-inhibitors such as sunitinib or sorafenib based on the presence of this SNP. The limited sample size when stratifying by treatment arms could explain these results.

Three SNPs were found relevant at predicting survival in advanced RCC patients. One of them in the Interleukin 8 (IL8) gene (rs2227543) is a 3 prime UTR variant, and therefore variations in these regions could significantly impact in the metabolism of the protein. IL-8 is a pro-inflammatory chemokine that execute an angiogenic function, thus, variations on this gene could influence tumor cell growth and angiogenesis. Only one report has associated this SNP with cancer, suggesting a potential role of genetic variations in IL genes as predictors of shorter DFS and OS in colorectal tumors. [34]. Likewise in our series, the presence of this genetic variant was associated with shorter OS.

The other two SNPs relevant in the advanced cohort (rs9800958 and rs2302273) are located in PRKAR1B, an oncogene related with cell growth and PDGFRB respectively. Both demonstrated a protective effect in our series with longer OS for the patients that harbour these variants. Rs9800958 is an intron variant of PRKAR1B and rs2302273 is located in the 5 prime UTR variant of PDGFRB and therefore could affect the gene product by altering the binding of the transcription factor [35]. However, no data about the precise role of these SNPs in cancer has been communicated yet.

When looking at prediction of response SNPs in the VEGFA gene resulted of interest. The polymorphism rs699947 predicted worse prognosis in our analysis. This variant has been evaluated in metastatic RCC by other groups with contradictory results. In some series appears as a positive prognostic factor [28, 36, 37] while others deny its prognostic or predictive value [19, 38]. In the same gene, the presence of rs3025010 in our series was associated to worse prognosis. There are only two oncology reports about this SNP, one in non-small cell lung cancer [39] and other in hepatocellular carcinoma [40] but neither of them established any correlation between the SNP and the response rate.

On the other hand, the presence of rs594942 in VEGFB has been associated with better response in our series. We have found only one citation of this polymorphism in metastatic colorectal cancer but without significance in the study [41].

All these results show the variability on the interpretation of polymorphisms depending on the type of cancer or the populations where they are evaluated. Nevertheless, the present exploratory study identified a set of SNPs that could improve prognostic and predictive estimation in RCC patients. Yet, the study might have a number of limitations that need to be taken into account. First the treatment varied across patients, although the majority (86%) received tyrosine kinase inhibitors targeting VEGFR/PDGR. This fact could compromise the real predictive value of these genetic variants. Another limitation of the study is the multiple testing. In a relatively small cohort of patients, multiple SNPs (variables) are evaluated. Therefore, these results need to be cautiously interpreted and require further validation in larger series. Yet, the data here presented are hypothesis generating and could eventually help in optimizing patient selection in cancer therapeutics and improve prognostic estimation through genetic characterization.

## MATERIALS AND METHODS

### Selection and characteristics of patients

Patients with localized and advanced RCC treated in the University Hospital “Virgen del Rocío” in the period 2000–2013 were included in the study. Paraffin embedded tumor samples were collected and patients were divided in two cohorts: those with localized disease and those with advanced RCC. The study protocol was approved by the Ethic Committee of Biomedical Investigation of Andalucía and conducted according to the principles of the Declaration of Helsinki.

The following inclusion criteria were considered: histologically confirmed diagnosis of primary RCC, complete clinical information and adequate tissue available (60%–75%). As clinical data the following were included: sex, age, date of diagnosis, TNM stage, histological type, tumor differentiation (Furhman grade), surgical treatment (partial or complete nephrectomy), systemic treatment (tyrosine kinase inhibitor (TKI) or mammalian target of rapamycin (mTOR) inhibitor, grade 3 or 4 toxicities, date of last visit or death and cause of death. All patients were treated following clinical guidelines and scientific evidence. Objective response was classified according to RECIST 1.1 as complete response (CR), partial response (PR), stable disease (SD), or progression of disease (PD).

### Selection of SNPs involved in angiogenesis and/or metabolism of antiangiogenic drugs

Sixty-four SNPs in 18 genes involved in angiogenesis and the mechanism of action of the drugs utilized in RCC therapeutics were selected (VEGFA, VEGFB, VEGFC, VEGFR2, VEGFR3, PRKAR1B, PDGFB, PDGFRA, PDGFRB, ABCB1, ABCG2, NR1/2, NR1/3, CYP3A4, CYP3A5, VHL, IL-8, IL-6) (Table 4). The selection of the SNPs to be analyzed was not systematic. Given the particular biology of RCC and the drugs utilized for treatment of this cancer we first selected genes involved in angiogenesis and also those related to the mechanism of action of tyrosine kinase inhibitors targeting VEGFR/PDGFR. Additionally we took into consideration previous studies, allele frequency in European and Iberian population (reference 100 Genomes Project), Hardy-Weinberg Equilibrium (Genotype frequencies are determined by allele frequencies at that locus) and linkage disequilibrium between SNPs determined by Haploview v4.2 software. This can be perceived as a limitation of the study [42]. Indeterminate results were coded as missing values for statistical analysis.

**Table 4:** SNPs in genes related with angiogenesis or metabolism of antiangiogenics in RCC

GENE	SNP							
**VEGFA**	rs699947	rs833061	rs3025010	rs3025033	rs2010963	rs1570360	rs3025039	
**VEGFB**	rs4930152	rs594942						
**VEGFC**	rs2016110	rs1485766	rs11947611	rs2877967	rs4604006			
**VEGFR2**	rs2305948	rs1870377	rs12505758	rs10013228	rs11941492	rs2071559	rs1531290	rs6828477
**VEGFR3**	rs307826	rs307821	rs6877011					
**PRKAR1B**	rs9800958	rs9768991						
**PDGFB**	rs9611117	rs879180						
**PDGFRA**	rs35597368	rs2114039	rs6554162	rs1800812	rs4358459	rs2228230	rs17739921	
**PDGFRB**	rs246395	rs246394	rs3816018	rs17708574	rs2302273	rs3828610	rs2304060	rs17656204
	rs11748255	rs11740355	rs3776081	rs4324662				
**ABCB1**	rs1045642	rs1128503	rs2032582					
**ABCG2**	rs2231142							
**NR1/2**	rs3814055	rs2276707						
**NR1/3**	rs2307424	rs4073054						
**CYP3A4**	rs2740574							
**CYP3A5**	rs776746							
**VHL**	rs779805	rs1642742						
**IL-8**	rs2227543	rs4073						
**IL-6**	rs1800795							

### DNA isolation and quantification

Paraffin embedded samples from patients with RCC were obtained from surgical specimens from nephrectomy. For each sample of 10 µm, paraffin was removed and DNA was isolated with DNA kit QiAGEN protocol. DNA concentration was determined by Nanodrop (Thermo Scientific, DE, USA).

### Amplification of the samples and TaqMan SNP assays

DNA was amplified, mixing 20 ng/µl of each DNA with the PreAmp Master Mix and PreAmp Pool (Life Technologies, Madrid, Spain) in 96-plate wells. This plate was sealed with MicroAmp clear adhesive film, centrifuged 30 s and put into GeneAmp PCR System 9700 that is specifically designed for the amplification of nucleic acids using the Polymerase Chain Reaction (PCR) process. The PCR conditions were: hold 95°C, 10 min; denature 95°C, 15 sec, 16 cycles and anneal/extend 60°C, 4 min, 16 cycles. Afterwards, each sample was diluted (1/20) with buffer TE (Tris-EDTA). The plate could be used immediately or kept at –20°C until next day. Samples were transferred duplicated into microArrays by a robotic axis. The result were obtained and interpreted by the TaqMan Genotyper Software (Life Technologies).

### Statistical analysis

The primary objective in the localized tumors cohort was to correlate the presence of SNPs with a worse DFS and OS. DFS was defined as the time between the diagnosis and the date of a radiological progression or death and OS as the time between the diagnosis and the date of death or last date of follow-up.

In the metastatic patients cohort overall RR, PFS and OS were analyzed and correlated with the presence SNPs. We considered overall RR as the percentage of CR and PR. The PFS was defined as the interval between the first day on systemic treatment and the date of radiological PD or death. Overall survival was defined as the time between the first day on treatment and the date of death or last date of follow-up.

Descriptive statistics were used to define the most relevant clinical features. The chi-squared test or Fisher’s exact test were used in order to know the most relevant clinical variables to be included in the multivariate analysis. For this purpose, the DFS, PFS and OS parameters and RR variables with *p* < 0.25 or those considered clinically relevant based on the previous literature on RCC were selected. These characteristics were: for patients with localized disease: type of nephrectomy (partial/complete), Furhman Grade (3–4), TNM stage and for patients with metastatic disease: Furhman Grade (3–4), TNM stage prognosis group (favorable vs intermediate/poor), metastasis lung and/or bones, Karnofsky, hemoglobin, time between nephrectomy and systemic treatment. ECOG was not included because the low number of patients in localized disease. All SNPs were tested in a univariant analysis for association with DFS, PFS and OS using Kaplan-Meier statistics and in a multivariate analysis using Cox proportional hazards to know the association between the presence of each SNPs and survival adjusting for potential confounding factors. Patients who had not progressed at database closure were censored at last follow-up. Also a chi-squared and a logistic regression were used to compare the presence of the SNPs and worse RR and the association of grade 3–4 toxicity with the presence of certain SNPs. *P <* 0.05 was considered significant. All analyses were performed using the Statistical Package for the Social Sciences software (SPSS 20.0 for Windows; SPSS Inc, Chicago, IL).

## Abbreviations

RCC: Renal Cell Cancer
SNP: Single Nucleotide Polymorphism
OS: Overall Survival
DFS: Disease Free Survival
PFS: Progression Free Survival
RR: Response Rate
ccRCC: clear cell Renal Cell Cancer
VHL: Von Hippel -Lindau
VHLp: Von Hippel -Lindau product
HIF: Hypoxia Inducible Factors
PDGF: platelet-derived growth factor
VEGF: vascular endothelial growth factor
PD-1: program-death 1
MET: hepatocyte growth factor receptor
MAF: minor allele frequencies
IL8: Interleukin 8
TKI: tyrosine kinase inhibitor
mTOR: mammalian target of rapamycin
CR: complete response
PR: partial response
SD: stable disease
PD: progression of disease
LD: linkage disequilibrium
